# Don’t cry over spilt milk: a review on consumer-based corporate reputation in private ophthalmology services

**Published:** 2018

**Authors:** Consuela-Mădălina Gheorghe, Victor Lorin Purcărea, Iuliana-Raluca Gheorghe

**Affiliations:** * “Carol Davila” University of Medicine and Pharmacy, Bucharest, Romania

**Keywords:** consumer-based corporate reputation, private ophthalmology services, experiential marketing, sustainability

## Abstract

A growing interest has been identified in defining and measuring corporate reputation in both business and academia. Monitoring and managing the intangible assets of an organization is an important matter and the most investigated intangible asset is reputation. In healthcare services, and implicitly, in ophthalmology services, corporate reputation has generated many controversial debates, due to their characteristics such as intangibility, variability, perishability, and inseparability. Reputation can be defined as a synthesis of opinions, perceptions, and attitudes of an organization’s stakeholders. In ophthalmology services, corporate management experts are more aware of the importance of corporate reputation and its implications on an organization’s performance. Consumers of private ophthalmology services are the primarily generators of revenues and they place great value on their health and minimize the perceived risks of the services by trusting a healthcare organization’s reputation. Consequently, ophthalmology services have been characterized by high-involvement and emotional vulnerability. Hence, consumer-based reputation in the context of private ophthalmology services encompasses affective responses, cognitive responses, the past experiences of consumers as well as their behavioral intentions. However, consumer-based corporate reputation is the sum of the existing consumer’s perceived reputation and the potential consumer’s perceived reputation. In ophthalmology services, corporate reputation experts recommend building a reputation focused on experiential sustainability, which is very difficult to achieve as it implies responsibility, frugality and sacrifice.

## Introduction

Over the last decade, there has been a growing interest in defining and measuring corporate reputation in both business and academia. The loss of confidence of investors, analysts, consumers and other stakeholders has been acknowledged to be potentially harmful for any sustainable business on the long term [**1**]. Thus, monitoring and managing the intangible assets, such as the reputation of an organization, is of important matter. In a simple approach, an organization’s reputation is a reflection of how it is regarded by its stakeholders. In a more complex approach, building a strong reputation reveals the degree of trust and credibility of society in the organization’s services, and, in turn, reputation helps an organization achieve its goals and objectives [**1**]. Along with society’s development, the role of an organization’s societal responsibilities has evolved as well, the orientation of strategies being switched from gaining profit to community responsibility [**1**]. 

This vast palette of responsibilities defines a multidimensional reputation when organizations deal with hostile environments, in time of crises by guaranteeing goodwill, and, lastly, it may be a competitive advantage [**1**].

In healthcare services, and implicitly, in ophthalmology services, corporate reputation has generated many controversial debates, due to their characteristics such as intangibility, variability, perishability, and inseparability [**2**]. Moreover, ophthalmology services present high levels of trustworthiness and reliability, healthcare consumers being unable to determine their technical features, and, so they take into consideration other functional service aspects, as for instance, the reputation and image of an organization [**3**].

**The corporate reputation conceptualization**

Increased competition in a globalized economy pledges for the management of intangible assets of an organization in the shape of reputation and image, which would lead to a sustainable competitive advantage [**4**]. 

The academic interest in corporate reputation has derived from the branding literature and the earlier literature on organizational identity [**1**]. Further, corporate reputation is a term closely linked to stakeholder theory and it has been mostly described in the academic literature as a perceptual representation or assessment of an organization by its different stakeholders and by its different social expectations that people attribute to it [**1**].

From an organizational perspective, corporate reputation brings a core advantage as it provides the opportunity of reducing transactional costs and positively influences both financial and consumer outcome variables such as consumer trust, satisfaction and loyalty [**4**]. However, recent literature acknowledged that corporate reputation is the most critical, strategic and enduring intangible asset of an organization [**4**]. In addition, Weiwei stated that since 1990s, reputation has been perceived as a valuable strategic organizational resource [**5**]. 

As a dynamic concept, corporate reputation develops if information about an organization’s achievements and activities spread out and, at the same time, several interactions occur between the organization and its stakeholders. Hence, corporate reputation is built over time, offering a relatively more stable and enduring status than image [**1**]. Different stakeholders, as suppliers, salesmen, competitors, consumers, investors, employees and local communities, may have different perceptions regarding the organization, based on their cultural and social backgrounds, as well as the information delivered [**1**]. Moreover, the vast majority of stakeholders do not assess their perceptions by interacting in a direct manner with the organization, but through third-party sources as in mass media or listening to opinion leaders [**1**]. 

Monitoring and controlling information may turn out to be challenging for most organizations because reputation is influenced by a variety of outside sources, in fact, being a collective phenomenon or a synthesis of opinions, perceptions and attitudes, which is made up of both cognitive and affective dimensions [**1**]. Further, Fombrun stated that corporate reputation is a “perceptual representation of a company’s past actions and future prospects that describe the firm’s appeal to all of its key constituents” [**6**]. 

Although corporate reputation is built on external and internal stakeholders’ perceptions, it may easily be confused with organizational identity and organizational image, as illustrated in **Table 1**. Moreover, organizational identity is built inside the organization, deriving from an organization’s culture (current practices, history, values and organizational behavior) and respectively, organizational image which is built inside the external stakeholders’ minds, in the shape of temporal direct and indirect perceptions of experiences [**1**]. Still, corporate reputation may be investigated as both image and identity, if it is expressed by one type of stakeholder [**1**].

**Table 1:** Differences between organizational identity, organizational image, and corporate reputation

	Organizational Identity	Organizational Image	Corporate Reputation
Stakeholders: Internal or external	Internal	External	Internal and external
Perceptions: Actual or desired	Actual	Desired	Actual
Emanating from inside or outside the organization	Inside	Inside	Inside and outside
Positive or negative perception of the possible organization	Positive or negative	Positive	Positive or negative
Relevant question	“Who/ what we believe we are?”	“What/ who do we want others to think we are?”	“What are we seen to be?”
Source: Percy Marquina Feldman, Rolando Arellano Bahamonde, Isabelle Velasquez Bellido. A new approach for measuring corporate reputation. RAE. Sao Paolo. Jan-fev 2014; 54(1):53-66.			

A positive corporate reputation also brings forth a competitive spirit and many other advantages, as for instance, it improves the consumer’s perception of the quality of services, increases sales and positive word-of-mouth, improves the capacity of hiring and retailing qualified personnel in organizations, raises the motivations and morale of employees, protects the value of the organization by diminishing the impact of scrutinizing, crisis and competitive attack, establishes a better market position, allows access to cheaper capital [**1**]. 

**Fig. 1:**
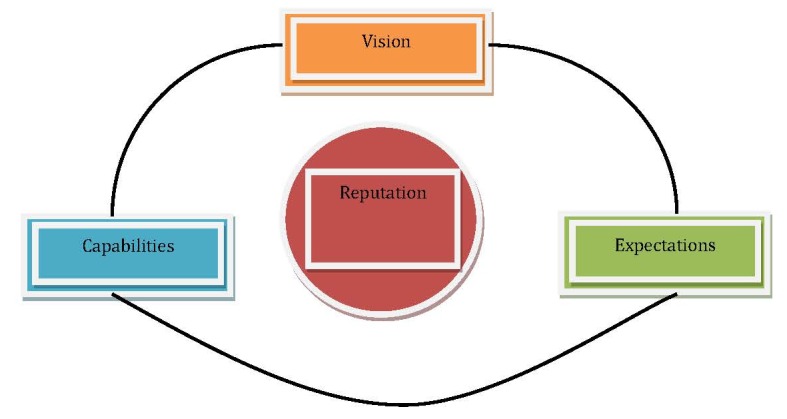
The strategic elements of an organization’s reputation

In order to configure an ideal strategy for managing corporate reputation, an organization should have the capacity to diagnose its weak and strong components, segmented on stakeholders [**1**]. As such, reputation management is an active, focused, and scientific approach in communicating with stakeholders. An efficient reputation management consists of the following interconnected elements, being aligned in the same directional strategy: vision, capabilities and expectations (**Fig. 1**). 

*The Vision component* refers to what the organization desires to be and how it wants to be seen, whereas *The capabilities component* suggests what makes the organization different from its competition and builds its strategy accordingly. Apart from these components, *The expectations component* means how stakeholders perceive the organization and, respectively, what is its position in their minds [**1**]. 

Moreover, by identifying both the internal and the external alignments of different stakeholders, reputation is actually measured. Reputation gaps between the internal and external stakeholders have immediate consequences on the long-term, leading to the loss of trust, downfall of profit and a wrongful orientation of the organization [**1**]. On the other hand, while taking into consideration these components, an organization may focus on implementing strategies to reinforce its current position in comparison to the competition, so as to have a constant and integrated overlooking on the alignment of vision, capabilities and expectations. 

**Corporate reputation in ophthalmology services**

In ophthalmology services, corporate management experts are more aware of the importance of corporate reputation and its implications on an organization’s performance. 

In industries, such as ophthalmology services, where competition is tough, periodic evaluations of general performances as well as success ratios have received attention in the regional and global competition. Weigelt and Camerer emphasized the need to measure the performance of an organization by its reputation, which “plays a strategically important role in service markets such as experiential services, where the pre-purchase evaluation of service quality is vague or partial” [**1**]. More exactly, as ophthalmology services are intangible, experts will integrate the reputation of the organization in the operational delivery process of services [**1**]. Further, in this respect, the ophthalmology organizations, which provide quality and reliable services, will gain favorable corporate reputation, which, in turn, will have an impact on the linkage between the consumers and the organization, based on trust. 

Although most corporate reputation specialists elaborate their strategies around the established relationship between corporate financial performance and corporate reputation, as it is perceived by managers and executives, we assume that in ophthalmology services, in order to assess an in-depth picture, all strategies should be consumer oriented.

Consumers of private ophthalmology services are, primarily, generators of revenue and, secondly, they should be segmented and investigated as existing consumers and potential consumers; their collective attitudes form the consumer-based reputation of an ophthalmology organization [**7**]. 

Moreover, the level of perceived risk, which comes along with the ophthalmology delivery of the service, has a significant effect on the relationship established between consumer-based reputation and non-monetary costs such as loyalty and positive word-of-mouth [**7**]. Although consumers use corporate reputation as a first-order screening criterion, it should be managed accordingly, suggesting proper identification of the types of risks involved, such as performance, physical, financial, psychological, social and temporal risks [**8**,**9**]. Performance risk relates to the consumers’ perception that a purchased service would not raise to their expectations and outcomes, and, at the same time, organizations being in the incapacity of delivering the promised benefits whereas the physical risk refers to the overall appearance of the threats of the service delivery. Further, financial risk is linked to the actual costs as well as to the incidental costs that encompass the stress of unpredictable situations. The psychological risk includes both individual perceived risks, in the shape of cognitive dissonance, of not making the right choice and the societal risks, when the individual’s peers disagree with his selection. Still, temporal risk is the most common specified risk an individual may perceive when related to healthcare services. Generally, temporal risk describes the amount of time required to receive the service, the time spent in case of a service failure, travelling time, waiting time, the speed of service delivery, the opening hours. However, given the nature of the healthcare market, which is a complex social phenomenon, it cannot be assumed that corporate reputation is formed similar to other industries [**7**]. As such, individuals place great value on their health and minimize their stakes by trusting the reputation of a healthcare organization. In addition, healthcare services, and, implicitly ophthalmology services, have been characterized by high-involvement and emotional vulnerability of consumers [**7**]. Yet, not identifying the antecedents of an ophthalmology organization’s reputation may have as outcomes reduced consumer retention, low market share and profitability [**7**]. Thus, the antecedents of consumer-based reputation in the context of ophthalmology services are affective responses, cognitive responses, the past experiences of consumers as well as their behavioral intentions [**7**]. 

Using the classification of consumers into existing consumers and potential consumers, corporate reputation specialists would implement consumer-based reputation strategies in a more effective manner. 

Consumer-based reputation management in the context of ophthalmology services

a) Existing consumers

**Fig. 2:**
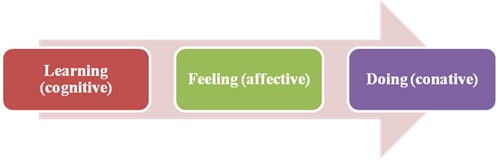
Alternative 1 to Effects Hierarchy Model in ophthalmology services

**Fig. 3:**
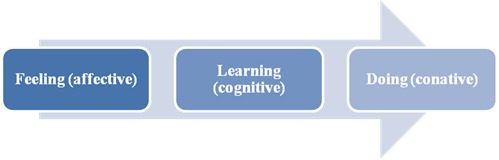
Alternative 2 to Effects Hierarchy Model in ophthalmology services

For existing consumers, experts may use strategies that focus on the informative and remembering functions of the Marketing Communications. Marketing Communications are the means by which a supplier of services, values, and ideas represents itself to its target audience with the goal to encourage the service experience by consumers. From the earliest days of consumer research, it was acknowledged that the consumer needs, require support when making decisions prior to purchase. Many consumers judge a source of information according to three characteristics: credibility, attractiveness and power [**10**]. The source credibility reflects how much confidence a receiver of information has in the source, the source attractiveness encompasses the degree of the source’s attractiveness and persuasion perceived by the receiver and, respectively, the source power is linked to the compliance of the receiver with the request involving a real or perceived reward. From an organization’s perspective, marketing communications have been effectively implemented if one of the hierarchies of effects models was adopted. The most known models are AIDA and DAGMAR [**11**]. The traditional framework of any hierarchy model consists of messages that should determine primarily a cognitive (thinking) effect, followed by an affective (feeling) effect and, finally, a conative (doing) effect [**12**]. Cognition is the “mental activity” of an individual shaped by knowledge, beliefs, or thoughts whereas the affective component consists of feelings and emotions, and conation reflects the intention or the behavior of an individual to perform an activity. Further, the AIDA model explains how the persuasive communication works, suggesting attention, interest, desire, and action [**13**]. In a healthcare context, the cognitive component focuses on increasing the attention and interest of consumers, the affective component encompasses the desire to act in a certain way and the conative component deals with the decision whether to take action or not. However, referring to a more practical approach, an alternative effects hierarchy model must be considered in the context of ophthalmology services. The sequences of stages an ophthalmology consumer may follow are shown in **Fig. 2** and **Fig. 3** [**11**].

After identifying the sequence of stages, a communications manager may elaborate strategies to raise the reputation by investigating the flow, channels and information needs as well as communication impacts on the motivation and performance of ophthalmology consumers.

b. Potential consumers

The objective of every ophthalmology organization is to attract potential consumers and the most effective method to achieve it, is by implementing experiential marketing strategies.

Experiential marketing is the process of identifying and satisfying consumer needs and desires in a win-win situation by ensuring benefit to potential consumers. More exactly, experiential marketing is defined by the assumption that strategies should not concentrate only on the supply but rather on the consumption and experience processes shaped by designing an entire “theatre” of a gradual operational process. The strategic experiential modules (SEMs) encompass several dimensions: the sense, feel, think, act, and relate [**14**]. In ophthalmology services, it has been concluded that feel experience, think experience and act experience influence the intention of a consumer to purchase the service [**15**]. 

As the blueprint of the experiential marketing strategic modules may be replicated easily, corporate reputation experts recommend the building of a reputation focusing on sustainability, which is very difficult to achieve as it implies responsibility, frugality and sacrifice. The common element in both experiential marketing and sustainability is that in the pursuit of sustainability, healthcare services may be pleasant experiences and not a joyless sacrifice. As such, experiential sustainability is made up of the following modules [**16**]:

a. Sensory sustainability reflects the delivery of services by attracting consumers through sensory appeals such as vision, touch, taste, scent and sound. In ophthalmology services, experts may use unique designs of the operational delivery process of services, play certain music, as well as spread around a specific scent. Moreover, an unforgettable experience may be provided in virtual reality.

b. Affective sustainability is linked to the marketing messages that invoke strong emotions and feelings within consumers. In ophthalmology services, managers may inspire strong emotional experiences in giving free eye consultations to disadvantaged populations or raise funds for eyeglasses and donate them to children in need. 

c. Behavioral sustainability includes measures of recycling plastic materials and protecting the environment. Further, in ophthalmology services, the organizations may minimize the use of plastic instruments in their activities. 

d. Intellectual sustainability is illustrated in terms of how a sustainable context may potentially stimulate the intellectual curiosity of the consumers. 

Nevertheless, the sustainable experiences may include one or more of the four experiential sustainable modules, as they are interrelated. When consumers achieve a positive, hedonic and fun-filled experience when going to an ophthalmologic consultation as well as behave in a sustainable manner, it will increase the quality of life and wellbeing of the consumer and encourage the implementation of positive collective value-driven sustainable behaviors so as to raise the consumer-based corporate reputation and market share of the ophthalmology organization and also increase the competitive advantage in the marketplace [**17**]. 

## Conclusions

The corporate reputation of an organization is now considered a key variable in improving the organization’s attractiveness and its capacity for retention of both consumers and investors [**1**].

A consumer-based corporate reputation is vital for an ophthalmology organization because it helps maintain the existing consumers and attract and gain loyalty of potential consumers by adopting the principles of the AIDA model for the former and the experiential sustainability strategies for the latter. 

According to Fombrun, an organization builds a strong corporate reputation if they concentrate on [**6**]:

- distinctiveness

- focus

- consistency

- identity

- transparency

Moreover, in the ophthalmology context, there are a few elements organizations should consider when building their consumer-based reputation [**18**]:

- responsibility – ophthalmology organizations should support worthy causes related to environmental responsibility as well as societal responsibility;

- communications ophthalmology organizations should address their consumers in a fully disclosed manner by implementing an integrated marketing communications plan. 

- services – in creating and delivering ophthalmology services, organizations should offer high quality and innovative services, which would generate consumer satisfaction and positive word-of-mouth.

- financial measures – ophthalmology organizations emphasize in their communications messages that they are doing better financially than their competition by making different investments in the field.

- leadership - the ophthalmology organization’s managers have solid leadership knowledge and implement good governance practices.

To conclude, the building blocks of consumer-based corporate reputation should include emotional appeals, vision and leadership, social responsibility strategies, financial performance and quality services [**18**].

**Fig. 4:**
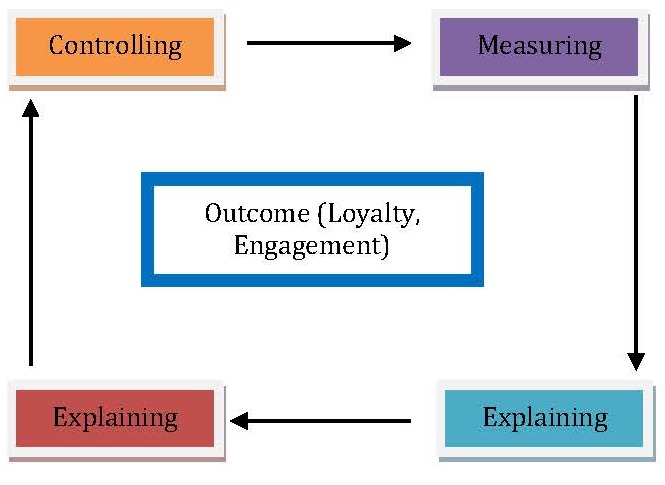
The consumer-based reputation management cycle for existing consumers of a private ophthalmology organization (Source: [**18**], p. 79)

Classifying consumers into existing consumers and potential consumers helps ophthalmology organizations elaborate appropriate strategies. However, a starting point for ophthalmology managers consists of which decisional outcomes should be followed. For existing consumers, ophthalmology organizations should conceive their growing reputation plan on raising loyalty and management by following the stages illustrated in **Fig. 4**.

The Reputation Management cycle enables managers to identify the drivers which have the most significant impact on their outcomes.

**Fig. 5:**
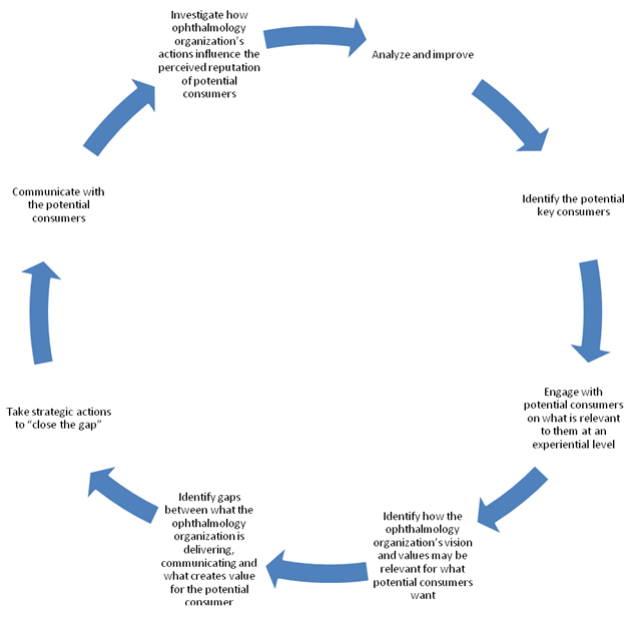
Experiential Sustainable Reputation Management Cycle for potential consumers of private ophthalmology organizations (source: adapted from [**18**], p. 101).

For potential consumers, a private ophthalmology organization should implement an experiential sustainability plan in order to raise its reputation. At an operating level, the challenging task reside in engaging consumers and include their inputs into the design and delivery management of services, taking a rather holistic approach in setting directions and shaping actions. **Fig. 5** describes The Experiential Sustainable Reputation Management Cycle which would improve the reputation building of an ophthalmology organization in the minds of consumers.
